# Heterogeneity in Pure Microbial Systems: Experimental Measurements and Modeling

**DOI:** 10.3389/fmicb.2017.01813

**Published:** 2017-09-20

**Authors:** Rebeca González-Cabaleiro, Anca M. Mitchell, Wendy Smith, Anil Wipat, Irina D. Ofiţeru

**Affiliations:** ^1^School of Engineering, Chemical Engineering, Newcastle University Newcastle upon Tyne, United Kingdom; ^2^Interdisciplinary Computing and Complex BioSystems (ICOS), School of Computing Newcastle University, Newcastle upon Tyne, United Kingdom

**Keywords:** population heterogeneity, single cell analysis, flow cytometry, population balance models, individual based models

## Abstract

Cellular heterogeneity influences bioprocess performance in ways that until date are not completely elucidated. In order to account for this phenomenon in the design and operation of bioprocesses, reliable analytical and mathematical descriptions are required. We present an overview of the single cell analysis, and the mathematical modeling frameworks that have potential to be used in bioprocess control and optimization, in particular for microbial processes. In order to be suitable for bioprocess monitoring, experimental methods need to be high throughput and to require relatively short processing time. One such method used successfully under dynamic conditions is flow cytometry. Population balance and individual based models are suitable modeling options, the latter one having in particular a good potential to integrate the various data collected through experimentation. This will be highly beneficial for appropriate process design and scale up as a more rigorous approach may prevent *a priori* unwanted performance losses. It will also help progressing synthetic biology applications to industrial scale.

## Introduction

Microbial populations developing in seemingly homogenous environments have been historically considered as formed by identical individuals. In reality no two cells in a pure culture are alike, even if they are derived from single clonal colonies (Ackermann, 2015). This phenomenon is of fundamental importance in biotechnological fermentations as the yields obtained will be lower if the cells are not in the same optimal productive state (Fernandes et al., 2011).

In bioprocess industries the bioreactors, and in particular the stirred tanks, are the central production units. The performance of any bioreactor is the emergent property of the activity and interactions at the single cell level and therefore, variations at this level can profoundly affect the dynamics and productivity of the process. Fluctuations that affect cell metabolism in industrial fermentations are estimated to generate losses of 30% or above (Lara et al., 2006; Takors, 2012). Moreover, sometimes recombinant protein production processes fail completely for reasons which are not fully understood but can be related to heterogeneity in the microbial population (Rosano and Ceccarelli, 2014).

The effect of cell individuality when using bacteria for obtaining useful products has been emphasized recently in several publications (Li and You, 2013; Wyre and Overton, 2014a,b; Chen et al., 2015). Nevertheless, the true impact of microbial population heterogeneity on bioprocesses remains unknown (Delvigne and Goffin, 2014) and therefore it is not systematically considered in design. This is partially due to the fact that experimental biological data obtained with traditional methods represents population average information (Pasotti and Zucca, 2014) which means that the performance of individuals is masked (Ackermann and Schreiber, 2015). Another potential reason is the relatively limited options for monitoring the heterogeneity under dynamic conditions. There are suggestions that minor subpopulations will not have significant influence on the whole population function (Lidstrom and Konopka, 2010), but more recent work emphasizes that non-genetic variation plays an important role in the overall biosynthetic performance of a bioprocess (Xiao et al., 2016).

Ultimately, industry needs to be able to engineer heterogeneity to obtain better yields and more robust processes. This requires both quantitative evaluation of the change of individual cells in time and of their interaction with the environment (Bley, 2011; Sauer and Mattanovich, 2012). Furthermore, this information needs to be included in mathematical frameworks used for design and control in order to have a realistic representation of the bioprocesses and to improve their performance.

**FIGURE 1:**
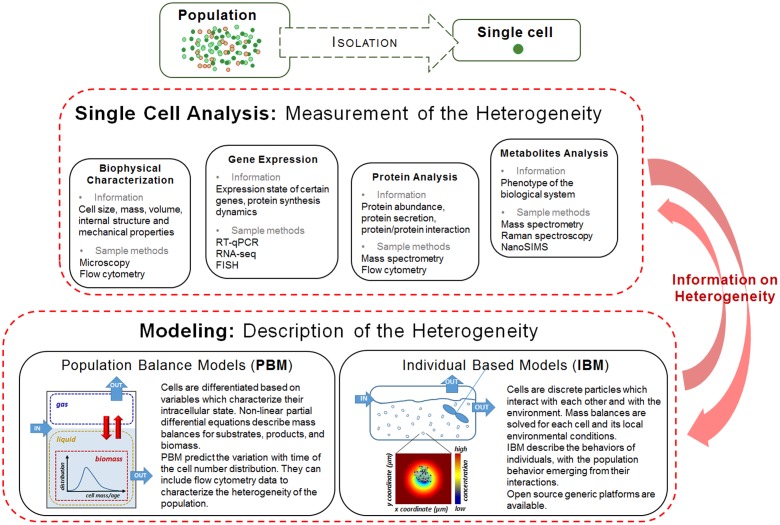
Single cell analysis methods and modeling approaches for characterizing population heterogeneity. An individual or a group of sorted individuals isolated from a cell population can be characterized with respect to its biophysical properties, gene expression, protein and metabolite characteristics. The data collected through experimentation is then included in mathematical models which will help interpreting it and further inform the bioprocess design. Reciprocally, the experimental data will help validate the mathematical models proposed.

In this mini-review we present an overview of the experimental methods used for characterizing the cell to cell variation in bacterial cultures and the corresponding mathematical tools for modeling them (see **Figure 1**), with a focus on the appropriate ones for fermentation processes.

## Sources of Cell Heterogeneity

The sources of heterogeneity in clonal microbial populations are biological (intrinsic) or environmental (extrinsic), or both. Whereas the intrinsic heterogeneity is generated by factors as cell cycle states, age distribution or the stochasticity of gene expression and metabolic reactions, the source for the extrinsic heterogeneity are the fluctuations in the environment. Therefore the latter represents a physiological response to stress (Lidstrom and Konopka, 2010; Ryall et al., 2012) and a survival strategy developed over evolutionary times (Booth, 2002; Sumner and Avery, 2002). This is the real challenge in the scaling-up of bioprocesses as poor mixing and heat transfer limitations generate concentration gradients which further influence the cells physiology.

A proposed way to overcome extrinsic heterogeneity and obtain similar performance in large scale reactors compared with laboratory reactors is to use strains specifically engineered to withstand certain environmental variability (Löffler et al., 2016). However, some investigations, both by modeling (Lavric and Graham, 2010) and experimental studies (Chi Fru et al., 2011; Ofiţeru et al., 2012) suggest that bacterial populations display constant heterogeneity in apparently steady growth and habitat conditions, questioning the very existence of truly homogenous cultures (Grote et al., 2015).

## Experimental Methods

The first step in single cell analysis is the isolation and/or immobilization of individuals from cell suspension. The experimental methods employed for this include serial dilutions (the traditional method), physical trapping (mechanical, hydrodynamic or dielectrophoretic), flow suspension [e.g., flow cytometry and in particular fluorescence-activated cell sorting (Winson and Davey, 2000)] and micromanipulation [mechanical or with optical tweezers (Landry et al., 2013)]. Between these techniques, the use of trapping of single cells in lab-on-a-chip microfluidic devices is expanding.

Once the isolation is achieved, single cell heterogeneity can be assessed. Bioprocess monitoring requires high throughput methods which allow rapid and highly parallel experimentation, with relative fast processing time. In general, the methods for single cell analysis were primarily developed for basic research and not all of them are adapted to be used for fermentation processes (Geiler-Samerotte et al., 2013). At the same time, some micro tools for isolation and interrogation of single cells developed for mammalian cells need further refinement when dealing with smaller microbial cells (Love et al., 2013).

The experimental methods for monitoring and assessing single cell heterogeneity can be classified as: (i) biophysical characterization; (ii) gene expression; (iii) protein analysis; and (iv) metabolite analysis. Several detailed reviews exist on single cell heterogeneity analysis (Brehm-Stecher and Johnson, 2004; Amantonico et al., 2010; Fernandes et al., 2011; Lecault et al., 2012; Shi et al., 2015; Vasdekis et al., 2015). We are presenting a general overview, emphasizing the ones appropriate for microbial population under dynamic process conditions.

Biophysical characterization gives information on the cell size, mass, volume, internal structure, and mechanical properties. Optical microscopy is widely used, being the simplest and quickest method but with low throughput. A high throughput method which provides information on cell size is flow cytometry. The composition of the individual cells can be obtained by Raman spectroscopy, a label free optical method that has been used for bioprocess investigation (Huang et al., 2004).

Gene expression methods (e.g., RT-qPCR, RNA-seq, FISH) give information on the expression state of a certain gene and protein synthesis dynamics. However, if a method involves lysis of cells, the dynamics of the gene expression in bioprocesses cannot be followed. An alternative is to use reporter systems (e.g., green fluorescent protein and its variants) that can be monitored with fluorescent time-lapse microscopy (Young et al., 2012). The limitation in this case is that production cultures usually do not contain fluorescent protein as marker and therefore this method is less suitable for monitoring fermentation processes.

Protein analysis at the single cell level can, in theory, provide information on protein abundance, protein secretion, or protein/protein interaction. Flow cytometry is the most commonly used method for measuring the protein content (Wu and Singh, 2012). Mass spectrometry has a high sensitivity and offers high quantity of information, from simultaneous identification of proteins to their posttranslational modifications. A workflow for sorted subpopulations, involving flow cytometry and mass spectrometry, was reported by Jahn et al. (2013). Nevertheless, there are still significant limitations due to the complexity of the proteome, the small amount of protein and the various types of measurement to be performed.

Metabolites analysis (intracellular and extracellular) is an indirect measurement of the phenotype of the biological system. The small size of the microbial cell and the minute quantity of metabolites make their detection at the single cell level very challenging. Methods used successfully in proof-of-concept experiments are Raman microspectroscopy, secondary ion mass spectrometry (SIMS) and Fourier transform infrared spectroscopy (Heinemann and Zenobi, 2011; Armitage et al., 2013; Rubakhin et al., 2013). Coupling a microfluidic unit to a mass spectrometer has the highest potential to deliver relevant data. NanoSIMS is a powerful tool for revealing element distribution in nanometer-scale resolution (Musat et al., 2012; Gao et al., 2016). However, the single cell metabolite analysis is considered to still be in its early stages.

From the reviewed methods, flow cytometry is the most suitable, relatively fast and user-friendly for measuring phenotypic single cell heterogeneity in bioprocesses and under dynamic conditions (Want et al., 2009; Muller and Nebe-von-Caron, 2010; Ambriz-Aviña et al., 2014; Delvigne and Goffin, 2014; Baert et al., 2016). Flow cytometry measures the distribution of a large variety of cellular parameters across a cell population by analyzing the light scattering and fluorescent signals of stained cells which flow in front of a powerful light source (e.g., a laser beam). Individual cells can be segregated based on their size, shape, intracellular properties, membrane potential, and variation in fluorescent signal. Because the large number of cells (tens of thousands) measured in a short processing time, flow cytometry offers statistically significant results and provides a quantitative measurement of heterogeneity in the sample, having the potential to identifying rare cell types (Shapiro, 2000; Davey and Winson, 2003). Recently, in combination with supervised machine learning techniques, flow cytometry was used also for single cell identification of populations in synthetic bacterial communities (Rubbens et al., 2017).

Nevertheless, the challenge of the high throughput methods is the amount of data generated, which requires rigorous quality control, together with sophisticated bioinformatics and statistics. Therefore, although automated flow-cytometry was expected to be implemented for real-time quality programs in factories (Hewitt et al., 1999; Díaz et al., 2010), to date single cell characterization is not routinely used in-process (Royle et al., 2013).

A complementary way to evaluate the microbial phenotypic heterogeneity under realistic bioprocess conditions is by employing scale down single cell micro-cultivation devices in which large scale reactors conditions are mimicked. Single cells in lab-on-a-chip microfluidic devices allow parallelization and high throughput experiments (Grünberger et al., 2014; Dusny and Schmid, 2015; Rosenthal et al., 2015; Oliveira et al., 2016), contributing to large-scale bioprocess improvement (Grünberger et al., 2012; Ladner et al., 2017). Sorting of different subpopulations of cells in order to understand the physiological responses in fluctuating microenvironments was also performed by microarray analysis (Hewitt et al., 2007).

## Modeling Options for Heterogeneous Populations

There is currently a gap between the new methods for single cell analysis and the availability of mathematical models which can integrate the data collected. But models are essential in the design and control of bioprocesses. Use of the complex information obtained by investigations of cells at genomic, transcriptomic, proteomic, and metabolomic level to predict bioprocesses is challenging and requires multidisciplinary analysis and significant computational efforts (Zhang et al., 2010).

The traditional classification of the mathematical models for cell populations is in unsegregated/segregated and unstructured/structured. The least complex is a model unstructured and unsegregated, which considers a homogenous population represented by an average unstructured cell, while the most complex is a model structured and segregated, which considers a heterogeneous population of structured cells (Song et al., 2014). The behavior of an average cell is representative only for a synchronous population (Noack et al., 2008), but for a heterogeneous population the model needs to include at least the segregation in the biophase.

Since their initial development, single cell models were seen as a promise for connecting the macroscopic bioreactor with the microscopic one, the cell (Shuler, 1999). Integrated multi-omics predictive models can inform biological discovery but their application is in its infancy (Brink et al., 2016; Kim et al., 2016). Some authors have attempted models which involve a laborious theoretical development to account for different sources of heterogeneity (Stamatakis and Zygourakis, 2010), though the same authors acknowledge them as being far too complex to lend themselves for practical application (Fredrickson and Mantzaris, 2002). Therefore, so far, the distributed properties measured within cell population are not integrated in a single modeling framework appropriate to be used in design, optimization and control of bioprocesses (Henson, 2003; Müller et al., 2010; Fernandes et al., 2011).

Here, we are presenting two options for modeling heterogeneity: population balance models (PBM) and individual based models (IBM). Both modeling approaches describe the variation in the population, but, while the PBM consider each fraction of the population as a continuous phase, in IBM the cells are discrete particles.

### Population Balance Models

In PBM cells are differentiated based on variables which characterize their intracellular state. Most commonly these variables are cell age or/and cell mass. The mass balances for substrates, biomass, and products are represented by non-linear and partial differential equations which have as independent variables time and the internal state of the cells. The different phases during the cell life cycle can be represented. The results obtained with PBM will predict the time variation of the cell number distribution, as resulted from growth and division.

Multidimensional PBM can be developed based on flow cytometry data (Fernandes et al., 2013; Ramkrishna and Singh, 2014). Biological heterogeneity in bioreactors was modeled by coupling a population balance model with a biokinetic model (Morchain et al., 2013) and later with a hydrodynamic model (Pigou and Morchain, 2015). One important limitation of PBM is that they are computationally demanding if they are represented more than one single internal state of the cells and this limits on line applications (Royle et al., 2013).

### Individual Based Models

In IBM the cells are discrete particles which interact with each other and with the environment. Microbial characteristics are described at single cell level. This allows the study of the system behavior as a result of the properties and performance of the individual components (Railsback and Grimm, 2012). However, it is not always possible to simulate all the individual cells of the system due to computing constraints and choices need to be made about the type of agents used (a cell or a cluster of cells or superindividual) and the level of detail for each of them.

In the last two decades IBM have gained popularity in microbiology (Ferrer et al., 2008; Schuler et al., 2011; Hellweger et al., 2016) due to rapid advancement in computational technologies and the development of specialized software. Open source generic platforms are now available (e.g., Sklar, 2007; Lardon et al., 2011; Rudge et al., 2012; Coakley et al., 2016). However, due to their complex structure IBM require more computing skills than other modeling approaches.

Both PBM and IBM approaches can be used for multispecies fermentation and can take into account the environmental heterogeneity in bioreactors (see *Coupling the scales*). However, PBM models explicitly the behavior of the population and can include only limited cell properties. They are also more restricted in representing stochastic processes as problems of closure may arise (Ramkrishna and Mahoney, 2002). Instead, IBM models the behavior of individuals, each having its own properties, with the population behavior emerging from their interactions. Therefore, it has a higher potential to integrate the detailed data generated with single cell analysis. At the same time, IBM offers a better representation of the stochastic processes, being able to describe the average fluctuations and not only the average behavior in a population.

### Coupling the Scales

The solution for PBM and IBM involve a numerical method using discrete time steps. In biological processes there is a wide range of relevant timescales, varying from nanoseconds to hours. The use of time steps in solving the mathematical models means that all the transformations which have a timescale smaller than the time step chosen for the numerical solution will only be approximated. Their influence on the state variables may then results as non-realistically high. Therefore, it is important to understand the effect of the approximations on the final output of the model and how it affects its predictive capabilities (Gameiro et al., 2016).

For a complete mathematical representation of the bioprocess, suitable to be used in scale-up and design applications, a two-way coupling between mass transfer, hydrodynamics, and biology is required (Wang et al., 2015; Morchain, 2017). These interactions are important as extracellular micro-heterogeneities may amplify the intracellular ones and place an upper limit on productivity and bioprocess reliability (Vasdekis et al., 2015). Local environmental conditions generated by flow streams affect the microbial metabolism and can be described by computational fluid dynamics (CFD). The Euler-Lagrange method represents the appropriate option for studying the impact of substrate gradients on the microbial metabolism in conjunction with the hydrodynamics (Lapin et al., 2004; Liu et al., 2016; Haringa et al., 2017; Kuschel et al., 2017). However, because of the high number of individual cells involved in a fermentation, it is not feasible to directly couple IBM with CFD at the large scale. One useful approach is using statistical emulators (metamodels), which extract the significant information from microscale and are computationally much faster (Wilkinson, 2009; Conti and O’Hagan, 2010). The advantage of an emulator over using a continuous model is that the former will not select *a priori* the information to be transmitted across scales but it will be based on a detailed mechanistic single cell model, representing therefore a simplified simulation strategy to calibrate multi-scale models. This approach was recently implemented by Oyebamiji et al. (2017) as an attempt to scale up a microbial system.

## Conclusion

In industrial setups there is a tradeoff between cellular growth and process robustness (Carlquist et al., 2012). Understanding and controlling cell heterogeneity at the single cell level will generate more robust and efficient bioprocesses, as, for example, it has been proven that it is not the highest biomass concentration, but higher proportion of viable cells which gives the best productivity (Want et al., 2009). Insights into bioprocesses at single cell level are expected to contribute also to the development of more accurate mathematical models that can be applied to the prediction and control of fermentative processes (Zhang et al., 2015). This will be highly beneficial as appropriate process and bioreactor design, able to prevent *a priori* unwanted performance losses, is still missing (Takors, 2012) and scaling up has a high degree of empiricism (Brognaux et al., 2013). IBM have the potential to integrate protein measurements with genomics, transcriptomics and metabolomics, and to predict the dynamics of the system across scales and in different environments (Hellweger et al., 2016), giving a better evaluation of the overall system performance.

This is relevant also for synthetic biology, a rapidly growing field which is limited by the lack of understanding on complex fluctuations in physiology and fitness of overall microbial populations (Cardinale and Arkin, 2012). Therefore connecting the single cell dynamics and heterogeneity of cell population with the bioreactor performance is a strategically important objective that is vital to the translation of systems and synthetic biology into an industrial reality.

## Author Contributions

All authors contributed to the writing of the manuscript. IO carried out the initial literature review and wrote the initial draft. RG-C provided insight relating to the mathematical modeling. AM and WS provided expertise relating the experimental methods. AW provided over-all guidance of the work and editing of the text.

## Conflict of Interest Statement

The authors declare that the research was conducted in the absence of any commercial or financial relationships that could be construed as a potential conflict of interest.
